# A novel amino acid metabolism-related gene risk signature for predicting prognosis in clear cell renal cell carcinoma

**DOI:** 10.3389/fonc.2022.1019949

**Published:** 2022-10-14

**Authors:** Jiaqi Su, Xi Tian, Zihao Zhang, Wenhao Xu, Aihetaimujiang Anwaier, Shiqi Ye, Shuxuan Zhu, Yue Wang, Guohai Shi, Yuanyuan Qu, Hailiang Zhang, Dingwei Ye

**Affiliations:** ^1^Department of Urology, Fudan University Shanghai Cancer Center, School of Life Sciences, Fudan University, Shanghai, China; ^2^Department of Oncology, Shanghai Medical College, Fudan University, Shanghai, China; ^3^Shanghai Genitourinary Cancer Institute, Shanghai, China

**Keywords:** clear cell renal cell carcinoma, amino acid metabolism, cuproptosis, risk signature, transmembrane protein 72

## Abstract

**Background:**

Renal cancer is one of the most lethal cancers because of its atypical symptoms and metastatic potential. The metabolism of amino acids and their derivatives is essential for cancer cell survival and proliferation. Thus, the construction of the amino acid metabolism-related risk signature might enhance the accuracy of the prognostic model and shed light on the treatments of renal cancers.

**Methods:**

RNA expression and clinical data were downloaded from Santa Cruz (UCSC) Xena, GEO, and ArrayExpress databases. The “DESeq2” package identified the differentially expressed genes. Univariate COX analysis selected prognostic genes related to the metabolism of amino acids. Patients were divided into two clusters using the “ConsensusClusterPlus” package, and the CIBERSORT, ESTIMATE methods were explored to assess the immune infiltrations. The LASSO regression analysis constructed a risk model which was evaluated the prediction accuracy in two independent cohorts. The genomic alterations and drug sensitivity of 18-LASSO-genes were assessed. The differentially expressed genes between two clusters were used to perform functional enrichment analysis and weighted gene co-expression network analysis (WGCNA). Furthermore, external validation of *TMEM72* expression was conducted in the FUSCC cohort containing 33 ccRCC patients.

**Results:**

The amino acid metabolism-related genes had significant correlations with prognosis. The patients in Cluster A demonstrated better survival, lower Treg cell proportion, higher ESTIMATE scores, and higher cuproptosis-related gene expressions. Amino acid metabolism-related genes with prognostic values were used to construct a risk model and patients in the low risk group were associated with improved outcomes. The Area Under Curve of the risk model was 0.801, 0.777, and 0.767 at the first, second, and third year respectively. The external validation cohort confirmed the stable prognostic value of the risk model. WGCNA identified four gene modules correlated with immune cell infiltrations and cuproptosis. We found that *TMEM72* was downregulated in tumors by using TCGA, GEO datasets (p<0.001) and the FUSCC cohort (p=0.002).

**Conclusion:**

Our study firstly constructed an 18 amino acid metabolism related signature to predict the prognosis in clear cell renal cell carcinoma. We also identified four potential gene modules potentially correlated with cuproptosis and identified *TMEM72* downregulation in ccRCC which deserved further studies.

## Introduction

Renal cell carcinoma is the third most frequent genitourinary tumor in urology (1). It is estimated that there are 79,000 new patients diagnosed with renal tumors and 13,920 related deaths in the United States in 2022 (1). Clear cell renal cell carcinoma (ccRCC) is the most common type of kidney malignant tumor. Due to the atypical clinical symptoms in the early stage of ccRCC, nearly 30% of patients are diagnosed with advanced ccRCC and usually have a worse prognosis (1). Although immunotherapy has made enormous progress in prolonging the survival of metastatic ccRCC patients and becomes the first line of advanced ccRCC (2), the efficacy still needs to be improved. Thus, it is urgent to develop novel reliable biomarkers to predict the prognosis, tumor progression, and drug sensitivity and to guide personal precision therapy.

Metabolism reprogramming that supports cancer cells proliferating at a high rate has been identified as a hallmark of cancer (3). The alteration in glucose metabolism was known as the “Warburg Effect” (4). Besides, amino acids, lipids, and nucleotides metabolic processes affected by glycolysis also demonstrated aberrant dysregulations due to complicated crosstalk within the tumor microenvironment (TME). Amino acid metabolism not only participates in the synthesis of proteins but also involves in the regulation of the proliferation of cancer cells (5). Glutamine, a metabolic substance second only to glucose, provides carbon and nitrogen for the biosynthesis of biomacromolecules that are required for rapid tumor growth. In addition, amino acid metabolism is also correlated with drug resistance (6), ferroptosis (7), epigenetic modifications (8), and so on. Cuproptosis is a new form of regulated cell death induced by copper (9). The underlying link and regulatory network between amino acid metabolism and cuproptosis might provide new therapeutic strategies. An increasing number of studies has confirmed the irreplaceable role of amino acid metabolism-related genes in tumorigenesis (10–12). For example, CB-839, the inhibitor of glutaminase (GLS) could exert strong anti-tumor effects alone or in combination with targeted therapy in ccRCC cell lines (13). The following clinical trial confirmed the anti-tumor effects of CB-839 (14). Nicotinamide N-Methyltransferase (NNMT) could catalyze methyl transfer from S-adenosyl methionine (SAM) to nicotinamide (NAM). In ccRCC, NNMT protein was higher in tumor tissue and ectopic expression of NNMT promoted cancer cell proliferation (15). Nevertheless, the integral analysis of amino acid metabolism-related gene set has not been systematically investigated in ccRCC.

In this study, we thoroughly performed systematic and profound investigations of amino acid metabolism-related genes in ccRCC. First, we identified the differentially expressed genes between tumor and normal tissue related to amino acid metabolism with prognostic value. Two different clusters with different amino acid metabolism characteristics showed significant differences in survival, immune cell infiltrations, and cuproptosis-related genes. Then, we constructed an 18-LASSO-genes risk model in TCGA cohort and validated it in E-MTAB-1980 cohort. To explore the differences between two clusters in survival, we explored the differentially expressed genes (DEGs) between two clusters. Functional enrichment analyses of these DEGs were conducted. Lastly, we identified four gene modules that showed associations with immune cell infiltrations and cuproptosis-related genes, and validated *TMEM72* expression in ccRCC patients. These discoveries might promote the development of precision treatment for ccRCC and expand new research strategies.

## Methods

### ccRCC samples from FUSCC cohort

The tumor samples and paired normal tissues of 33 patients with ccRCC from the Department of Urology at FUSCC (Shanghai, China) were collected during surgery and recruited for the studies. All patients consented to the examination and signed an informed consent form. The Helsinki Declaration II was followed in the design of the study and the testing techniques. The Fudan University Shanghai Cancer Center’s ethical committee approved the study methods utilized in this research (FUSCC, Shanghai, China).

### Data acquisition, processing and amino acid metabolism-related genes

The RNA expression and clinical data of primary ccRCC were downloaded from UCSC Xena (xenabrowser.net) (16). The FPKM (Fragments Per Kilobase of transcript per Million mapped reads) was converted to TPM (Transcripts Per Million) and subsequently normalized in the log(X+1) algorithm. E-MTAB-1980 was considered as an external validation cohort and downloaded from the ArrayExpress database (https://www.ebi.ac.uk/arrayexpress/). The GEO datasets (https://www.ncbi.nlm.nih.gov/geo/) including GSE40435, GSE53757, and GSE36859 cohorts were downloaded to analyze *TMEM72* mRNA expression in ccRCC. The normalized gene expression profiles were downloaded and annotated. Probes were averaged if the multiprobes were mapped to the same gene. After excluding the patients without follow-up data, there were 522 tumor samples, 71 normal samples in TCGA cohort, and 101 tumor samples in E-MTAB-1980. The amino acid metabolism genes of REACTOME_METABOLISM_OF_AMINO_ACIDS_AND_DERIVATIVES were obtained from the Molecular Signatures Database (MSigDB) (https://www.gsea-msigdb.org/gsea/msigdb). The list of the gene set was presented in **Table S1**. The cuproptosis-related genes were obtained from previous research (9).

### Differential gene expression and Univariate COX regression analysis

The normalized transcriptional profiles were used to find differentially expressed genes (DEGs) between tumor and normal tissue using “DESeq2” package (|LogFoldchange|>1 and p <0.05 ). The Protein-Protein interaction (PPI) network of the 100 DEGs were retrieved from the STRING database (https://string-db.org/) (17). The DEGs and the gene set of amino acid metabolism intersected genes that were subsequently performed to Univariate Cox regression analysis with p <0.05. In total, the prognostic DEGs related to amino acid metabolism (aamRDEG) were identified.

### Subcluster analysis

Based on the prognostic amino acid metabolism-related DEGs (aamRDEGs), “ConsensusClusterPlus” package divided ccRCC patients into two subclusters (reps=50; pItem=0.8; clusterAlg=“km”; distance=“Euclidean”). The survival analysis was applied using “survival” package. “CIBERSORT” and “ESTIMATE” packages were used to evaluate the immune cell infiltrations in KIRC. The LM22 was obtained from the CIBERSORT website (https://cibersort.stanford.edu/). Then, we compared the immune cells infiltrations and cuproptosis-related genes between subclusters.

### Construction of amino acid metabolism-related risk signature

The least absolute shrinkage and selection operator (LASSO) regression analysis was applied in aamRDEGs to establish a risk model using “glmnet” package. The smallest λ is chosen to construct the model to ensure accuracy of the risk siganture (λ_min_=18). Thus, an 18-LASSO-genes risk signature was constructed and the risk scores were calculated: Risk score=ΣCoefficients×LASSO-genes. To ensure the universality of our risk model, we selected the median of the risk score as the cutoff value. Then the patients were divided into 2 groups (high risk VS. low risk). The survival analysis was performed to compare the outcomes of these two groups. The Area Under Curve (AUC) of the ROC curves were calculated to evaluate the accuracy of the risk signature. The E-MTAB-1980 cohort served as an external validation cohort to confirm the stability and accuracy of the risk model in ccRCC patients.

### Clinicopathological features correlation analysis

The patients without complete clinicopathological information were excluded in this section analysis. After that, there were 245 ccRCC patients in KIRC cohort and 99 ccRCC patients in E-MTAB-1980 cohort. The heatmaps of two cohorts were plotted using “pheatmap” package. We next performed Univariate and Multivariate Cox regression analyses to evaluate the predictive value of the risk signature model. The correlations between risk score and clinical phenotypes were conducted to assess the changes of the risk score in the process of tumor progression. The Sankey picture was used to identify the distribution of patients using “ggalluvial” package.

### Pan-cancer analysis of the 18-LASSO-genes

The Gene Set Cancer Analysis (GSCA) (http://bioinfo.life.hust.edu.cn/GSCA/) database was an integrated platform for gene set cancer analysis (18). Single nucleotide variation (SNV), copy number variation (CNV), methylation, and drug sensitivity (Genomics of Drug Sensitivity in Cancer, GDSC, https://www.cancerrxgene.org/; Cancer Therapeutics Response Portal, CTRP, https://portals.broadinstitute.org/) of the 18-LASSO-genes were investigated in human cancers. The top ten most altered genes of SNV were presented in the oncoplot. Pearson correlation tests were applied and p<0.05 was considered statistically significant.

### Functional enrichment analysis

To investigate the underlying mechanism of different outcomes between two clusters (Cluster B VS. Cluster A). The differentially expressed genes (DEGs) between two clusters were identified (|LogFoldchange|>1 and p<0.05). The DEGs were subsequently performed Gene Ontology (GO) and Kyoto Encyclopedia of Genes and Genomes (KEGG) pathways analysis in the DAVID (https://david.ncifcrf.gov/) database. The functional enrichment results were filtered by p<0.05 and FDR<0.1. Gene set enrichment analysis (GSEA) was used to identify potential biological process (BP) (c5.go.bp.v7.5.symbols.gmt) and KEGG pathways (c2.cp.kegg.v7.5.symbols.gmt) of DEGs between two different clusters using GSEA 4.1.0 software. The GSEA results were filtered by p<0.05.

### Weighted gene co-expression network analysis

The DEGs between two clusters (Cluster B VS. Cluster A) of 522 patients from the KIRC cohort were used to construct a co-expression network using the “WGCNA” package. Then, a weighted adjacency matrix was constructed to identify correlation strength between nodes using a power function. After choosing the best soft power of 6, the adjacency matrix was transformed into a topological overlap matrix (TOM), and the corresponding dissimilarity (1-TOM) was calculated. The average linage hierarchical clustering was performed based on a TOM-based dissimilarity measure with at least ten genes dendrogram to identify similar gene modules. The result of the cluster tree was displayed in the plots. The Pearson correlations between gene modules and ESTIMATE scores and cuproptosis-related genes were investigated to detect underlying impacts on immune infiltrations and programmed cell death procedures. GeneMANIA (http://genemania.org/) was applied to explore the PPI network of the gene module. GO analysis of yellow and brown gene modules was applied to determine where these genes enriched in.

### Real-time quantitative PCR analysis

The total RNA of 33 patients was extracted using TRIzol reagent (Invitrogen Life Technologies, USA). The reverse transcription was performed using EZBioscience 4× EZscript Reverse Transcription Mix II (EZBioscience, USA). The Real-Time Quantitative PCR (RT-qPCR) experiment was conducted using EZBioscience 2× SYBR Green qPCR master mix (EZBioscience, USA) and detected using QuantStudio™ Real-Time PCR Software. All the experiments were conducted according to the manufacturer’s instructions. The primers for *TMEM72* were as follows: forward, 5′- AGG GGC CTA CTT TGT GGC T-3′ and reverse, 5′- TTC TCC CTT ACT CTG TCT GCC-3′. The *TMEM72* expressions were calculated relative to that of GAPDH. Each sample was repeated three times and the average value was performed. The *TMEM72* mRNA expression was determined as 2^-ΔCt^ = 2^-(Ct (^*^TMEM72^*^) - Ct(^*^GAPDH^*^))^. The Wilcox test was used for the comparisons of the means of tumor and normal tissue. In addition, *TMEM72* of renal cancer cell lines were downloaded from the Cancer Cell Line Encyclopedia (CCLE) (https://sites.broadinstitute.org/ccle). Expression charts were performed using GraphPad Prism 9.0.0 software.

### Validation of TMEM72 protein expression

The immunohistochemistry staining was obtained from the Human Protein Altas (https://www.proteinatlas.org) (19). The *TMEM72* protein expression levels of tumor and normal tissue were assessed by HPA039894 and HPA062907 independently. In addition, we utilized Proteomic Data Commons (PDC) (https://pdc.cancer.gov/pdc/) website to investigate *TMEM72* protein expression and prognostic value using CPTAC data. We also explored the correlation between *TMEM72* mRNA expression and protein expression.

### Statistical analysis

The analysis has been performed using R 4.1.1 software and R packages. The figures were produced by Adobe Illustrator (CC 2020). The distinctions between these two groups were analyzed using the Wilcoxon rank sum test. The correlation test was performed in the Pearson correlation algorithm and survival analysis was performed in the log-rank algorithm. All of the hypothetical tests had a significant p value of 0.05 and were two-sided.

## Results

### Identification of amino acid metabolism-related prognostic genes

**Figure 1:**
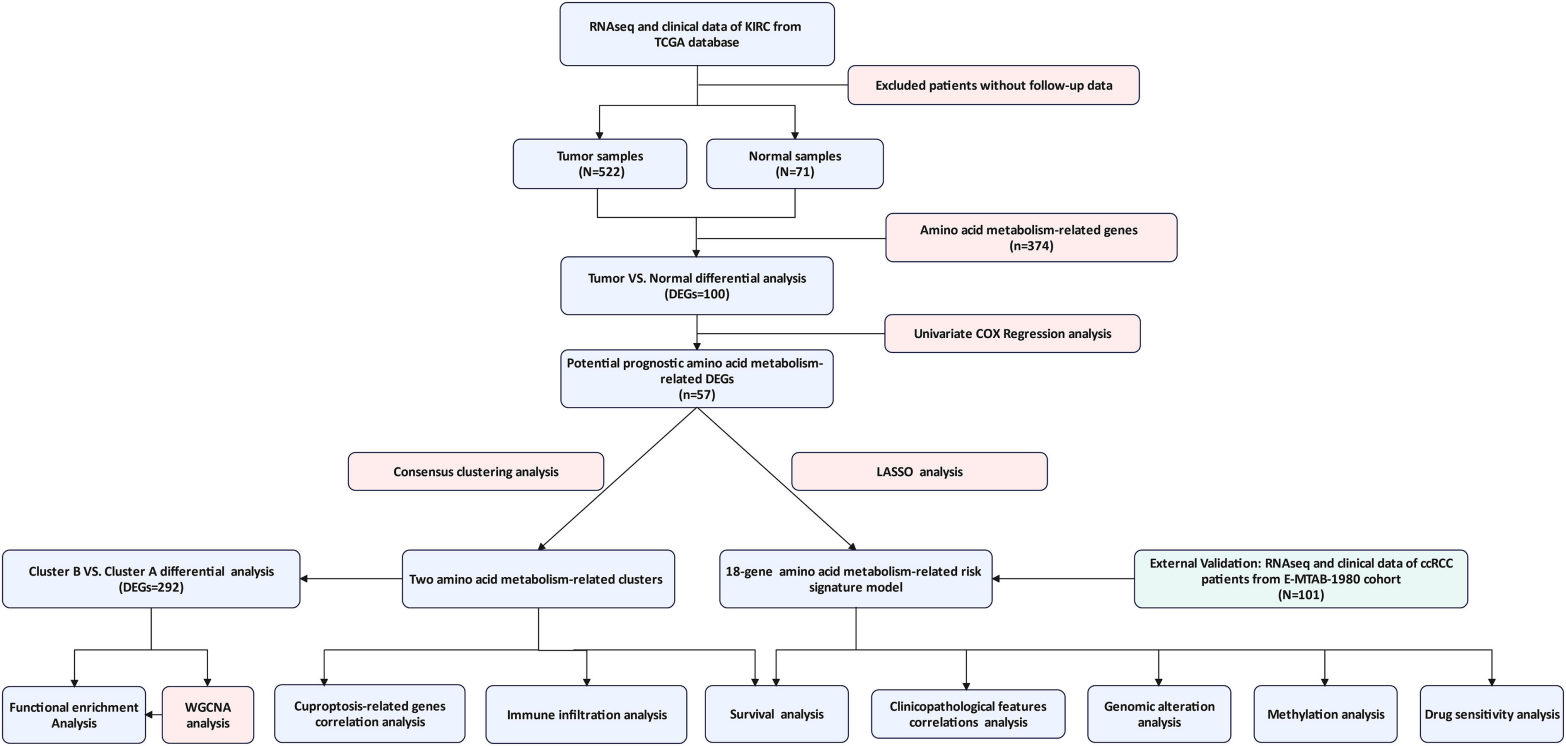
The complete workflow of the study. (TCGA, The Cancer Genome Atlas; KIRC, Kidney Renal Clear Cell Carcinoma; DEG, Differentially Expressed Gene; ccRCC, clear cell renal cell carcinoma; WGCNA, Weighted Gene Co-expression Network Analysis).

**Figure 2:**
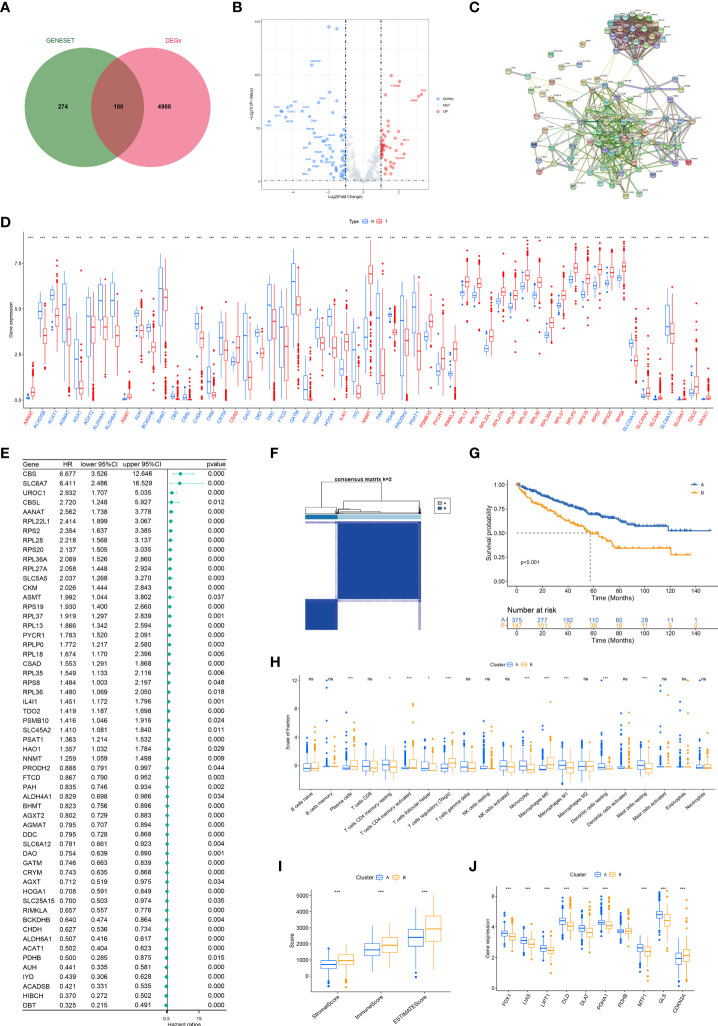
Consensus clustering of aamRDEGs and differences between two clusters. **(A)** The intersected genes of DEGs between tumor and normal tissue and gene set of metabolisms of amino acids. **(B)** The volcano plot of the 100 DEGs. **(C)** The PPI network of 100 DEGs. **(D)** The different expression levels of aamRDEGs between tumor and normal tissue. Red: upregulation in tumor. Blue: downregulation in tumor. **(E)** The HRs with their 95% CI of aamRDEGs with p<0.05 were conducted using Univariate Cox regression analysis. **(F)** TCGA cohort was divided into two clusters according to the consensus clustering (K=2). **(G)** The survival curve showed that Cluster A had a better prognosis compared to Cluster B (p<0.001). **(H, I)** The potential differences in immune cell infiltrations between two clusters were investigated by CIBERSORT and ESTIMATE. **(J)** The potential differences of cuproptosis-related genes between two clusters. ( ns, no significance; *,p < 0.05; **, P < 0.01; ***, P < 0.001) (DEG, differentially expressed gene; PPI, Protein-Protein Network; aamRDEG, amino acid metabolism-related gene; HR, HR, hazard ratio; CI, confidence interval).

The complete workflow of our study was present in **Figure 1**. The details of clinicopathological features of ccRCC patients in two cohorts were presented in **Table 1**. The DEGs between tumor and normal sample and amino acid metabolism gene set had one hundred intersected genes (**Figure 2A**). There were 57 genes upregulated and 43 genes downregulated (**Figure 2B**). The Protein-Protein interaction (PPI) network of the 100 DEGs were investigated from the STRING database to identify the protein interactions modelues and there were two main protein interactions modules (**Figure 2C**). The 57 prognostic aamRDEGs were obtained using Univariate Cox regression analysis. The expression and prognostic value of aamRDEGs were presented in **Figures 2D, E**. After Univariate Cox regression, 31 genes including *AANAT, ASMT, CBS, CBSL, CKM, CSAD, HAO1, IL4L1, NNMT, PSAT1, PSMB10, PYCR1, RPL13, RPL18, RPL22L1, RPL27A, RPL28, RPL35, RPL36, RPL36A, RPL37, RPLP0, RPS19, RPS2, RPS20, RPS8, SLC45A2, SLC5A5, SLC6A7, TDO2*, and *UROC1* demonstrated worse prognosis, while the other 26 genes were correlated with better outcomes in ccRCC patients (**Figure 2E**).

**Table 1:** Clinical phenotypes of the ccRCC patients from TCGA and E-MTAB-1980.

	TCGA cohort (%)	E-MTAB-1980 cohort (%)
Age		
<65	327 (62.64%)	52 (51.49%)
>=65	195 (37.36%)	49 (48.51%)
Gender		
Female	183 (35.06%)	24 (23.76%)
Male	339 (64.94%)	77 (76.24%)
T classification		
T1	268 (51.34%)	68 (67.33%)
T2	67 (12.84%)	11 (10.89%)
T3	176 (33.72%)	21 (20.79%)
T4	11 (2.11%)	1 (0.99%)
N classification		
N0	237 (45.40%)	94 (93.07%)
N1	16 (3.07%)	3 (2.97%)
N2	0	4 (3.96%)
NX	269 (51.53%)	0
M classification		
M0	412 (78.93%)	89 (88.12%)
M1	77 (14.75%)	12 (11.88%)
MX	33 (6.32%)	0
Stage		
Stage 1	262 (50.19%)	68 (67.33%)
Stage 2	56 (10.73%)	10 (9.90%)
Stage 3	120 (22.99%)	13 (12.87%)
Stage 4	81 (15.52%)	10 (9.90%)
Stage X	3 (0.57%)	0
Grade		
G1	14 (2.68%)	13 (12.87%)
G2	221 (42.34%)	59 (58.42%)
G3	205 (39.27%)	22 (21.78%)
G4	75 (14.37%)	5 (4.95%)
GX	7 (1.34%)	2 (1.98%)
Survival status		
Alive	351 (67.24%)	78 (77.23%)
Dead	171 (32.76%)	23 (22.77%)

TCGA, The Cancer Genome Atlas.

### Subclusters correlated with survival, immune infiltrations, and cuproptosis

The best k value (K=2) of the consensus analysis was selected from k=2 to k=9. Patients with ccRCC were classed into two subclusters according to the aamRDEGs expression. Cluster A had 375 patients and Cluster B had 147 patients (**Figure 2F**). Cluster A demonstrated a better prognosis compared to Cluster B with p<0.001 (**Figure 2G**). In addition, Cluster B showed a higher proportion of plasma cells, Tregs, and M0 macrophages than Cluster A (**Figure 2H**). Cluster B demonstrated higher StromalScore, ImmuneScore, and ESTIMATEScore compared to Cluster A (**Figure 2I**). Cuproptosis was a novel cell death induced by Cu^2+^ and correlated to mitochondria pathways (9). Amino acid seemed important for cancer cell’s metabolism and proliferation. Thus, we explored the potential differences between two clusters based on the amino acid metabolism-related genes. Of note, except for PDHB, eight genes including *FDX1, LIAS, LIPT1, DLD, DLAT, PDHA1, MTF1*, and *GLS* expressed higher in Cluster A, while *CDKN2A* expressed higher in Cluster B (**Figure 2J**). These data implied that the aamRDEGs had an underlying mechanism of regulating immune cell infiltrations and a potential correlation with cuproptosis, which might contribute to the worse outcomes.

### Construction and validation amino acid metabolism-related risk signature

**Figure 3:**
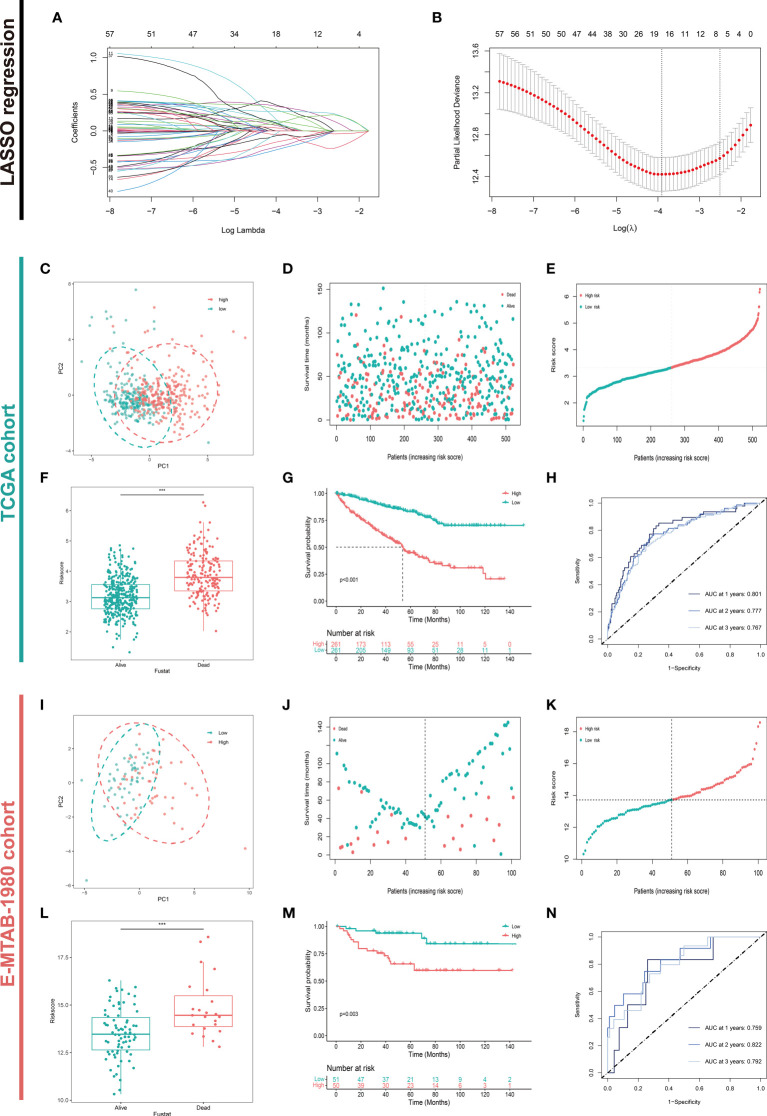
Construction and Validation of the Amino Acid Metabolism-Related Risk Signature. **(A, B)** Construction and validation of the candidate aamRDEGs using LASSO regression analysis and the λ_min_ =18 was selected. Validations of the risk signature in TCGA cohort were presented in **(C–H)**. **(C)** The distribution of two risk groups of TCGA cohort. **(D)** Survival status of the patients in TCGA cohort. **(E)** Ordered risk score in TCGA cohort. **(F)** The difference in risk score between live status. **(G)** Survival Analysis between two risk groups (p<0.001) (high risk VS. low risk). **(H)** The AUC of the ROC at the first, second, and third year in TCGA cohort. The validation of the risk signature in E-MTAB-1980 cohort were conducted in the same way and presented in **(I–N)**. (***P < 0.001) (aamRDEG, amino acid metabolism-related gene; LASSO, Least Absolute Shrinkage and Selection Operator; TCGA, The Cancer Genome Atlas; ROC, Receiver Operating Characteristic; AUC, Area Under Curve).

After LASSO COX regression analysis, the 18-LASSO-genes risk signature was established based on the λ_min_=18 (**Figures 3A, B**). The details of the risk score were presented in **Table 2**. The median value of the risk score was regarded as the cutoff value and divided patients into two different groups (high risk VS. low risk). The distribution of the patients was conducted in principal component analysis (PCA) (**Figure 3C**). The scatter diagrams of survival status and risk score of the KIRC cohort were presented in **Figures 3D, E**. Next, we explored the whether survival differences that existed between these two groups. The alive patients had a lower level of risk score (p<0.001) (**Figure 3F**). The survival analysis demonstrated that high risk group demonstrated a worse prognosis compared to low risk group (p<0.001) (**Figure 3G**). The AUC of ROC was 0.801, 0.777, and 0.767 at the first, second, and third year respectively (**Figure 3H**). The same analysis was performed in the E-MTAB-1980 cohort (**Figures 3I–N**). The results confirmed the stability and accuracy of the risk signature, which stressed the important role of the metabolism of amino acids in carcinogenesis.

**Table 2:** The coefficients of eighteen-LASSO-genes.

Gene	Coefficients
ACADSB	-0.0464985234558365
BCKDHB	0.0990744929210214
CBS	0.338169768953652
CSAD	0.328087925215322
HAO1	0.0636468902107542
HIBCH	-0.0601952071519661
HOGA1	-0.143534860112872
IL4I1	0.272026445233619
IYD	-0.245885162590596
NNMT	0.0969328934626091
PSAT1	0.133109807017969
PYCR1	0.265148205286679
RIMKLA	-0.210230274880309
RPL13	0.0853813702125398
RPL22L1	0.108528658183546
RPL36A	0.0373366566152849
SLC5A5	-0.000952564512580635
UROC1	0.0771235799115952

### Correlations between clinicopathological features and risk score

**Figure 4:**
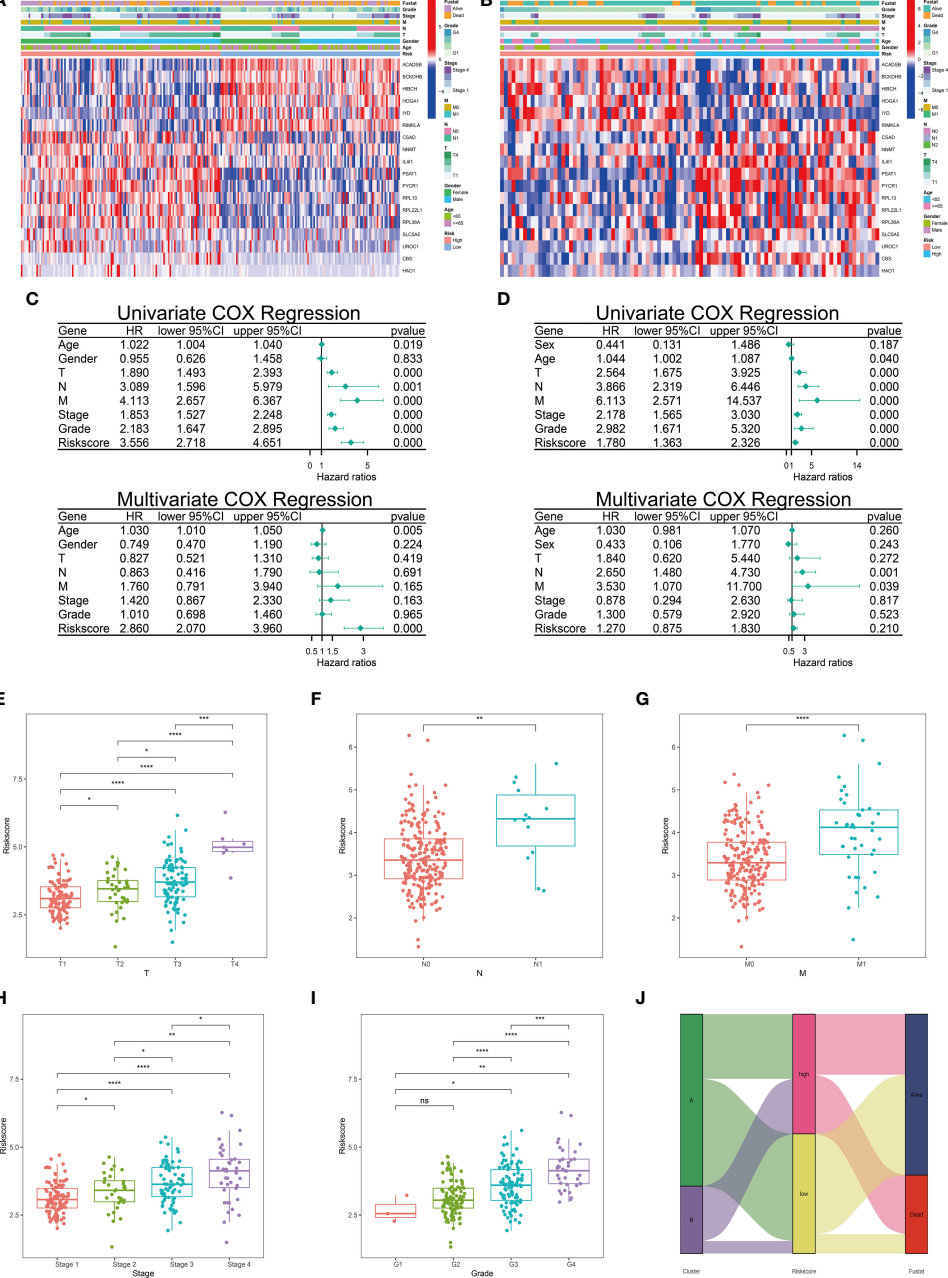
Correlations between clinicopathological features and risk score. The heatmap of different clinicopathological features and 18-LASSO-genes were conducted in TCGA cohort **(A)** and E-MTAB-1980 cohort **(B)**. Univariate and multivariate COX regression analyses of clinicopathological features and risk scores were conducted in TCGA cohort **(C)** and E-MTAB-1980 cohort **(D)**. **(E–I)** The correlations between clinical features and risk scores were conducted in TCGA cohort. **(J)** Distribution of different clusters and different risk groups in TCGA cohort. ( ns, no significance; *,p < 0.05; **, P < 0.01; ***, P < 0.001; ****, P < 0.0001) (TCGA, The Cancer Genome Atlas).

The patients with complete clinical phenotypes were incorporated in this section analysis. The clinicopathological features of 245 patients from KIRC cohort and 99 patients from E-MTAB-1980 cohort were presented in the heatmap (**Figures 4A, B**). Next, we explored the prognostic value of these clinicopathological features and risk scores. Univariate and Multivariate Cox regression analyses were performed in these two independent cohorts. In KIRC cohort, Univariate Cox analysis showed except for gender, the other indicators showed correlations with overall survival (OS) (HR>1, p<0.05). Only the risk score demonstrated independent ability to predict prognosis in the Multivariate Cox analysis (HR=2.860, p<0.001) (**Figure 4C**). The risk score was correlated with OS using Univariate Cox regression in the E-MTAB-1980 cohort, but could not be an independent indicator of prognosis according to the Multivariate COX analysis (**Figure 4D**). The inconsistencies could be attributed to the limited number of patients and other potential confounding factors. We next explored the risk score alterations in process of tumor progression. Risk score showed statistically significant positive correlations with T status, N status, M status, Stage status, and Grade status in the KIRC cohort (**Figure 4E–I**). The distribution of different clusters and different risk groups was plotted in **Figure 4J**. These results implied that the risk signature of metabolism of amino acids could be a stable and accurate model to predict prognosis and suggested amino acid metabolism-related genes had an underlying role in tumor progression.

### Pan-cancer analysis of 18-LASSO-genes

**Figure 5:**
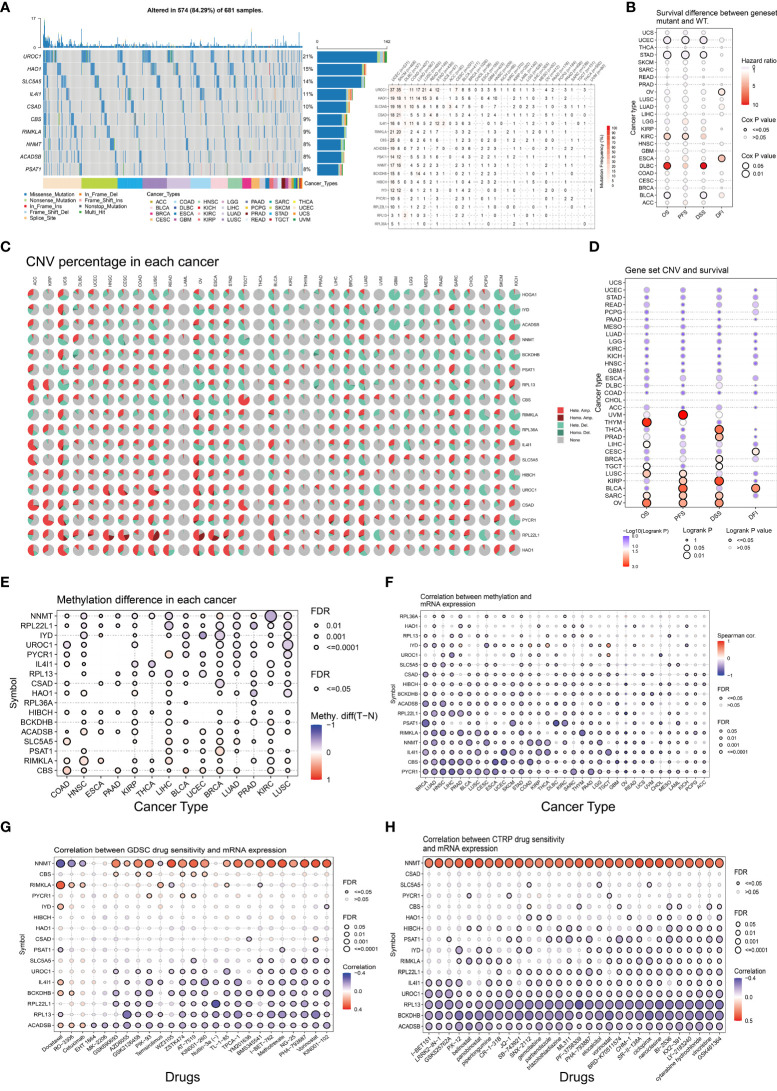
Pan-cancer analysis of 18-LASSO-genes. The pan-cancer analyses were conducted in the GSCA database. The summary of SNV of 18-LASSO-genes and correlations between SNV and survival were presented in **(A, B)**. The summary of CNV of 18-LASSO-genes and correlations between CNV and survival were presented in **(C, D)**. The degree of methylation and correlations between methylation degree and gene expression of 18-LASSO-gene were presented in **(E, F)**. The drug sensitivity of 18-LASSO-genes from GDSC and CTRP databases were presented in **(G, H)**. (SNV, single nucleotide variation; CNV, copy number variation; GDSC, Genomics of Drug Sensitivity in Cancer; CTRP, Cancer Therapeutics Response Portal) showe.

The pan-cancer analyses were conducted in the GSCA database. The top ten mutant genes and the frequency of 18-LASSO-genes were investigated in human cancers (**Figure 5A**). UROC1 (21%), HAO1 (15%), and SLC5A5 (14%) were the top three mutant genes in human cancers. The survival differences between18-LASSO-genes set mutant and wild type was found in UCEC (Uterine Corpus Endometrial Carcinoma), STAD (Stomach Adenocarcinoma), KIRC (Kidney Renal Clear Cell Carcinoma), DLBC(Lymphoid Neoplasm Diffuse Large B-cell Lymphoma), and BLCA (Bladder Urothelial Carcinoma) (**Figure 5B**). The summary of CNV and survival differences of the 18-LASSO-genes were investigated (**Figures 5C, D**). The methylation analysis demonstrated that the degree of methylation varied in different tumors. For example, *NNMT* showed a lower degree of methylation in tumors compared to normal tissue in KIRC, which could contribute to the high expression of *NNMT* in tumors (**Figures 5E, F**). The drug sensitivity from GDSC and CTRP database were presented (**Figures 5G, H**). *NNMT, UROC1, IL4l1, BCKDHB, RPL22L1, RPL13, and ACADSB* had statistically significant correlations with the IC50 of chemotherapeutic drugs, suggesting these genes could serve as potential therapeutic targets.

### Functional analysis of differentially expressed genes between two clusters

**Figure 6:**
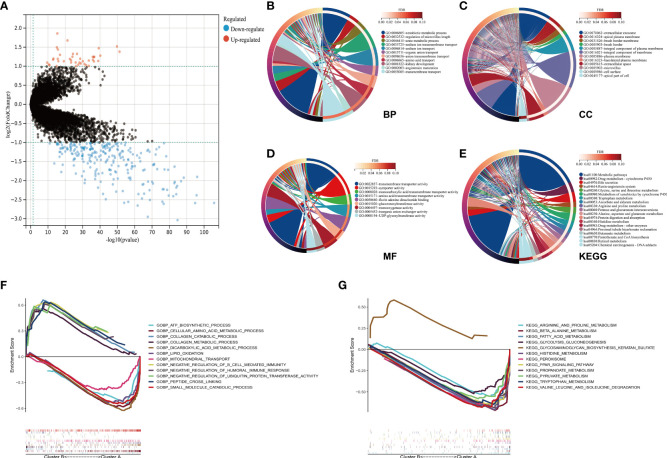
Functional analysis of DEGs between two clusters. **(A)** The 292 DEGs between Cluster B and Cluster A were presented in the volcano plot. **(B–E)** The functional enrichment results of 292 DEGs were conducted. Biological process in **(B)**; cellular component in **(C)**; molecular function in **(D)**; KEGG pathways in **(E). (F, G)** The GSEA results of two clusters were presented. (DEG, differentially expressed gene; KEGG, Kyoto Encyclopedia of Genes and Genomes; GSEA, Gene set enrichment analysis).

Obtained from the Sankey diagram (**Figure 4J**), we noted that the different clusters demonstrated close correlations with live status. To identify the key genes that contributed to the worse prognosis, we explored the differential expressed genes (DEGs) between two clusters, and the DEGs were presented in the volcano plot (Cluster B VS. Cluster A). There were 42 genes upregulated and 250 genes downregulated in Cluster B compared to Cluster A (**Figure 6A**). The function enrichment analysis of 292 DEGs demonstrated that biological process (BP) mainly enriched in xenobiotic metabolic process, regulation of microvillus length, urate metabolic process, and materials transport procession (**Figure 6B**); cellular component (CC) mainly enriched in extracellular exosome, and several membranes (**Figure 6C**); molecular function (MF) mainly enriched in transmembrane transporter activity, symporter activity, monocarboxylic acid transmembrane transporter activity, and amino acid transmembrane transporter activity (**Figure 6D**); KEGG pathways mainly enriched in metabolic pathways, drug metabolism-cytochrome P450, and metabolism of different amino acids (**Figure 6E**). The GSEA results demonstrated that Cluster B and Cluster A participated in different pathways, which might be correlated with the progression and prognosis of ccRCC (**Figures 6F, G**).

### Identification hub gene modules

**Figure 7:**
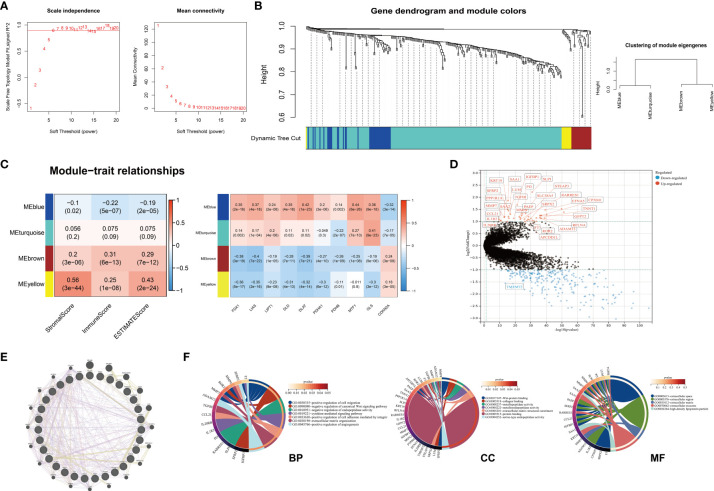
Identification of four hub gene modules. **(A)** Scale-free index analysis and mean connectivity analysis for selecting the best soft power. **(B)** Dendrogram of 292 DEGs obtained from 1-TOM (dissimilarity) into four MEs. **(C)** Heatmap of correlations between ESTIMATE scores, cuproptosis-related genes, and four MEs in KIRC. MEyellow and MEbrown were selected for the following analysis. **(D)** The expression levels of thirty-one genes from MEyellow and MEbrown (Cluster B VS. Cluster A) **(E)** The PPI network of 31 genes from MEyellow and MEbrown. **(F)** The GO enrichment analysis of thirty-one genes from MEyellow and MEbrown. (DEG, differentially expressed gene; TOM, topological overlap matrix; ME, module eigengene; KIRC, Kidney Renal Clear Cell Carcinoma; PPI, Protein-Protein Interaction GO, Gene Ontology).

To identify the co-expression gene modules from the DEGs obtained from different clusters, we performed the WGCNA analysis. The best soft power for WGCNA was selected as 6 (**Figure 7A**). Based on this, 292 DEGs were classed into four MEs (module eigengene) including MEblue, MEturquoise, MEbrown, MEyellow (**Figure 7B**). The MEbrown, MEyellow could be classified into one bigger gene module and MEblue, MEturquoise could be classified into the other gene module (**Figure 7B**). Because of the findings above, we explored the correlations between different gene modules and ESTIMATE scores, cuproptosis-related genes. Of note, MEblue gene module demonstrated negative correlations with ESTIMATEScores, while MEbrown and MEyellow demonstrated positive correlations with ESTIMATEScores (**Figure 7C**). Similar to the findings above, we found that cuproptosis-related except *CDKN2A* showed negative correlations with MEbrown and MEyellow gene modules (**Figure 7C**). MEblue and MEturquoise demonstrated contrary correlations. We next combine MEbrown and MEyellow as integrity to analyze their expressions. The results showed that there were 31 genes in total from MEbrown and MEyellow gene modules and most of the genes except *TMEM72* were higher in Cluster B (**Figure 7D**). To define the underlying mechanism that 31 genes might influence, we explored the PPI network from GeneMania and analyzed the functional enrichment (**Figures 7E, F**). Because of the limited gene number of the MEbrown and MEyellow modules, p<0.05 was considered significantly enriched. These genes enriched in positive regulation of cell migration, negative regulation of canonical Wnt signaling and so on in biological process, which implied the potential role in carcinogenesis (**Figure 7F**). The KEGG pathways demonstrated that the MEbrown and MEyellow module genes only enriched in the Wnt signaling pathway. The results above indicated that the genes of MEbrown and MEyellow modules might be central modules in tumor development and were associated with immune infiltrations and cuproptosis.

### *TMEM72* expression was downregulated in ccRCC

**Figure 8:**
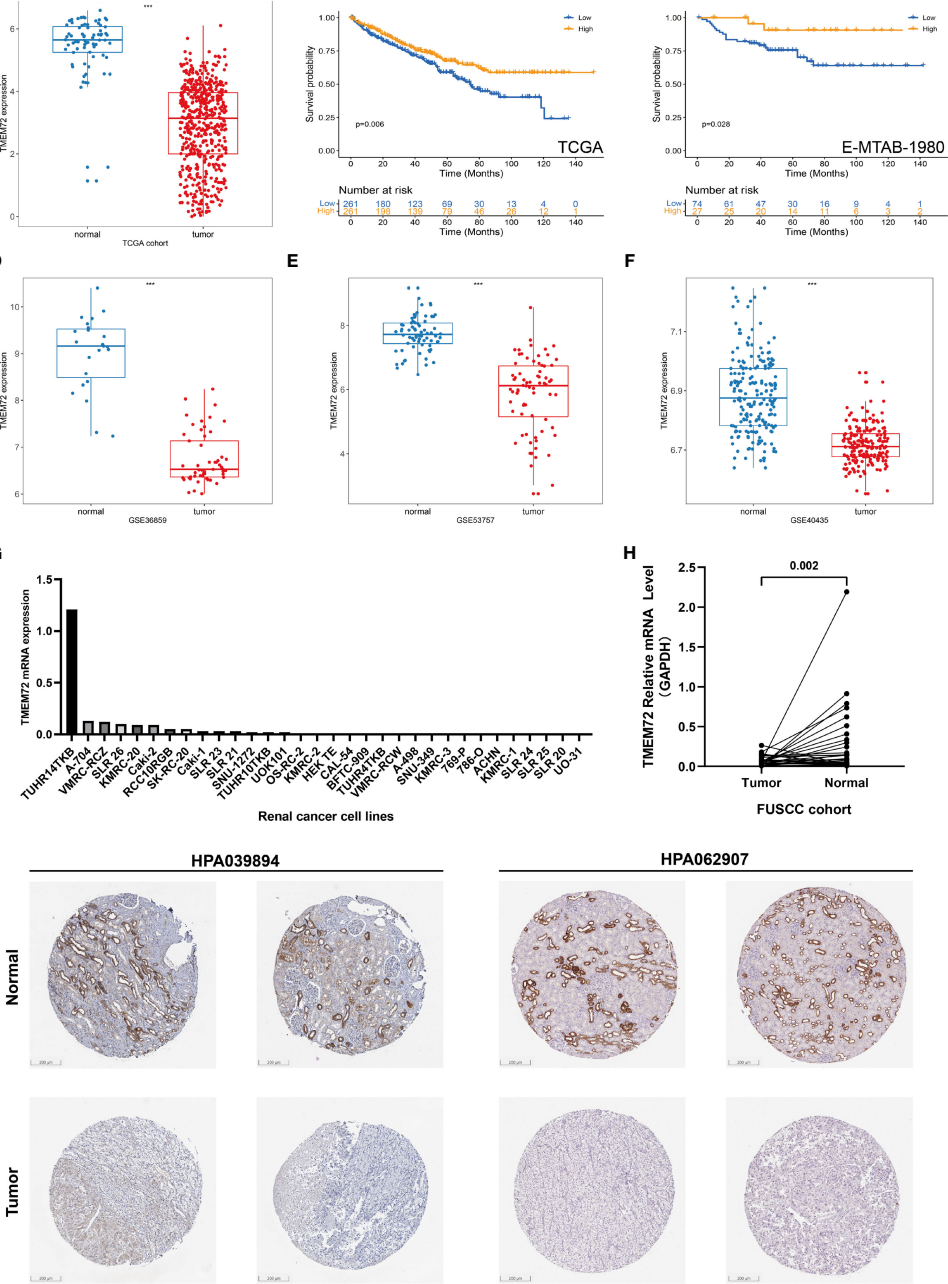
*TMEM72* expression in ccRCC. **(A)** *TMEM72* mRNA expression in KIRC cohort. **(B, C)** Survival curves suggested the prognostic value of *TMEM72* mRNA expression in KIRC and E-MTAB-1980 cohorts. **(D-F)** *TMEM72* mRNA expression in three external validation cohorts (GSE36859, GSE53757, GSE40435). **(G)** *TMEM72* mRNA expression in renal cancer cell lines. **(H)** *TMEM72* mRNA expression of 33 paired samples in FUSCC cohort. **(I)** Immunohistochemistry staining of *TMEM72* protein expression from HPA database. (***, P < 0.001) (KIRC, Kidney Renal Clear Cell Carcinoma; HPA, Human Protein Atlas).

Due to the specificity of *TMEM72* expression, we then explored the *TMEM72* expressions in TCGA, GEO, and the FUSCC cohorts. In TCGA, *TMEM72* was significantly downregulated in tumors compared to normal tissue (**Figure 8A**) and lower level of *TMEM72* had a worse prognosis in TCGA (p=0.006) and E-MTAB-1980 cohorts (p=0.028) (**Figures 8B, C**). Three external cohorts containing GSE40435, GSE53757, and GSE36859 confirmed that *TMEM72* expression was lower in tumors than in normal tissue (**Figures 8D–F**). The *TMEM72* mRNA expressions of renal cancer cell lines were presented in **Figure 8G**. In FUSCC cohort, *TMEM72* mRNA expression was higher in adjacent normal tissue than in tumors (p=0.002) (**Figure 8H**). Lastly, the immunohistochemical staining of *TMEM72* protein from the HPA database confirmed that *TMEM72* was downregulated in renal cancers at the protein level (**Figure 8I**). To further investigate *TMEM72* protein expression, we used PDC database to confirm our findings. As shown in the **Supplementary Figures 1A, B**, *TMEM72* protein expression was significantly decreased in ccRCC than in normal tissue. We next explored the parallel correlation between *TMEM72* mRNA and protein expression. The results demonstrated a highly correlated association between transcription and translation (R=0.8142) (**Supplementary Figure 1C**). Thus, we could infer the protein expression level from the RNA expression level, which was consistent with the above conclusion in our study. The survival curve demonstrated that patients with a higher level of *TMEM72* protein expression had a better prognosis compared to patients with a lower level of *TMEM72* protein expression (p=0.014) (**Supplementary Figure 1D**)

## Discussion

Renal cell carcinoma is one of the deadliest cancers around the world due to its high metastatic potential (20). With the advanced development of novel treatment strategies, however, the efficacy of immunotherapy and targeted therapy still needed to be improved. Hence, to better guide the treatments and predict the prognosis, novel biomarkers for patients with ccRCC were urgently in demand. The important role of amino acids is not only important for the anabolism of biomolecules needed by tumor cells but also for regulating the function integrality of immune cells in the tumor microenvironment (TME) which eventually contributes to the immune evasion (21, 22). For instance, Yang et al. confirmed the diagnostic value of three amino acids including glutamate in oral squamous cell carcinoma (23). Previous studies identified amino acid metabolism-related risk signatures to estimate the outcomes of patients with glioma (10, 24). Jiang et al. developed an amino acid catabolism-related gene signature associated with prognosis in pancreatic adenocarcinoma (25). However, the studies focusing on aberrant metabolism of amino acids still remained to be investigated in ccRCC, and the correlations between amino acid metabolism and immune cell infiltrations. In our research, we utilized TCGA cohort and E-MTAB-1980 cohort to investigate the prognostic value of amino acid metabolism-related risk signature, and explored the correlations with immune cell infiltrations and cuproptosis-related genes.

In total, there were 57 aamRDEGs with the prognostic value which divided ccRCC patients into two clusters with different amino acid metabolic traits. Cluster A correlated with better overall survival (OS), a lower proportion of Tregs cells, higher levels of Immunescore, Stromalscore, and ESTIMATEScore, and positively associated with cuproptosis-related genes except for CDKN2A and PDHB. To eliminate the effect of overfitting, the LASSO regression analysis finally constructed an eighteen amino acid metabolism-related risk signature. Risk scores were calculated and classified patients into two subgroups and correlated with patients’ outcomes. The high risk group showed a worse prognosis compared to the low risk group in TCGA cohort, which was confirmed in the E-MTAB-1980 cohort. Previous studies had verified the important role of NNMT in cancer progression. Proteomic analysis revealed that NNMT played an indispensable role in for the functional integrity of cancer associated fibroblasts (CAFs), which indicated the importance role of NNMT in regulating the stromal crosstalk in ovarian cancer (26). Thus, NNMT could serve as a potential therapeutic target (27). However, the literature related to the other LASSO genes in studying the underlying mechanism was limited. Hence, our study provided new perspectives to guide future research directions in the process of carcinogenesis and progression.

To explore the hub gene modules which contributed to the worse prognosis, the DEGs between two clusters were used to perform GO, and KEGG functional analysis. And the GSEA results revealed the different potential pathways or biological processes between these two clusters. Then WGCNA algorithm classified the 292 DEGs into four hub gene modules which showed different correlations with immune cell infiltrations and cuproptosis-related genes. We integrated MEyellow and MEbrown and found that only *TMEM72* from the 31 genes was downregulated in Cluster B when compared to Cluster A.

*TMEM72*, also known as *C10orf127* or *KSP37*, is located at 10q11.21 and belonged to the transmembrane proteins (TMEM) gene family. The TMEM protein family is one type of protein expressed on the cell surface like anchorage proteins or as structural proteins (28), but its specific functions were not yet been well investigated. A growing number of studies revealed that TMEMs were differentially expressed among human cancers, including *TMEM116* and *TMEM229A* in lung cancer (29, 30), *TMEM205* in hepatocellular carcinoma (31), *TMEM168* in glioblastoma (32), and *TMEM180* in colorectal cancer (33). In addition, the TMEM family also played an essential role in regulating the metastasis process and immune response in tumor progression (34). However, the literature about *TMEM72* was lacking. The previous study identified ten dysregulated TMEM family genes in ccRCC and found that *TMEM72* was significantly downregulated in tumors compared to adjacent normal tissue in ccRCC (p<0.01) (35). Consistent with the previous study, our study confirmed that *TMEM72* was downregulated in tumors than in normal tissue (p=0.002). What’s more, Wrzesiński et al. discovered that *TMEM72* expression levels decreased to a greater extent in metastases than in non-metastases, although no statistical difference was reached (39.78 fold VS. 5.13 fold, p=0.051) (35). Limited by the relevant research about *TMEM72*, it was hard to elucidate the reason for the *TMEM72* aberrant expression in tumor cells. Future studies should focus on the location and function of *TMEM72* protein, which might reveal the underlying mechanisms of tumor progression.

There were certain limitations to our research. First, there are no underlying mechanisms or crosstalk elucidated clearly by this study. Thus, future studies about mechanism investigations should be done. The correlations between amino acid metabolism and cuproptosis-related genes might help discover new therapeutic targets. Second, multicenter and large-cohort studies were necessary to verify the risk signature before applying it in clinical application. Third, with the development of new technologies, gene expression should be evaluated at the single-cell level. Lastly, the impact on immune cell infiltrations caused by amino acid metabolism differences should be investigated and studied in the future study.

In conclusion, we discovered 57 amino acid metabolism-related genes correlated to OS and firstly constructed an 18 amino acid metabolism-related risk signature for ccRCC prognosis prediction. In addition, we identified four gene modules that had significant impacts on patients’ survival. Our study was based on high-throughput data from TCGA and E-MTAB-1980 cohorts, provided a unique profound understanding of ccRCC prognosis and offered the theoretical groundwork for future investigations on amino acid-related novel therapeutic strategies.

## Data availability statement

The datasets presented in this study can be found in online repositories. The names of the repository/repositories and accession number(s) can be found in the article/**Supplementary Material**.

## Ethics statement

Written informed consent was obtained from the individual(s) for the publication of any potentially identifiable images or data included in this article.

## Author contributions

JS, XT, ZZ and WX were responsible for experimental design, experimental analysis and thesis writing. AA, YW, SY, SZ and GS were responsible for data screening, collection and writing. YQ, HZ and DY were responsible for the guidance and review of the thesis. All authors contributed to the article and approved the submitted version.

## Funding

National Key R&D Program of China (2019YFC1316005); Natural Science Foundation of Shanghai (20ZR1413100), the Natural Science Foundation of China (No. 82172817) and the Shanghai "Science and Tech-nology Innovation Action Plan" Medical Innovation Research Project (22Y11905100) for the financial support. This article has not received sponsorship for publication.

## Acknowledgments

The principal authors are very grateful for the data support provided by the TCGA, ArraryExpress and GEO databases.

## Conflict of interest

The authors declare that the research was conducted in the absence of any commercial or financial relationships that could be construed as a potential conflict of interest.

## Publisher’s note

All claims expressed in this article are solely those of the authors and do not necessarily represent those of their affiliated organizations, or those of the publisher, the editors and the reviewers. Any product that may be evaluated in this article, or claim that may be made by its manufacturer, is not guaranteed or endorsed by the publisher.

## Supplementary material

Supplementary Table 1 The gene list of REACTOME_METABOLISM_OF_AMINO_ACIDS_AND_DERIVATIVES.

Supplementary Figure 1 TMEM72 protein expression in CPTAC database. **(A)** The TMEM72 protein expression of paired samples. **(B)** The TMEM72 protein expression of unpaired samples. **(C)** The positive correlation between TMEM72 mRNA and protein expression. (R=0.8142) **(D)** The survival curve showed that downregulation of TMEM72 was a symbol of poor prognosis. (p=0.014).(CPTAC, Clinical Proteomic Tumor Analysis Consortium)

## Abbreviations

TMEM72, Transmembrane Protein 72; TME, Tumor Microenvironment; ccRCC, clear cell renal cell carcinoma; PPI, Protein-Protein Interaction; DEG, Differentially Expressed Gene; LASSO, Least Absolute Shrinkage and Selection Operator; GSCA, Gene Set Cancer Analysis; SNV, Single Nucleotide Variation; CNV, Copy Number Variation; GDSC, Genomics of Drug Sensitivity in Cancer; CTRP, Cancer Therapeutics Response Portal; GO, Gene Ontology; BP, Biological Process; CC, Cellular Component; MF, Molecular Function; KEGG, Kyoto Encyclopedia of Genes and Genomes; GSEA, Gene Set Enrichment Analysis; WGCNA, Weighted Gene Co-expression Network Analysis; FUSCC, Fudan University Shanghai Cancer Center; TCGA, the Cancer Genome Atlas; ESTIMATE, Estimation of Stromal and Immune cells in Malignant Tumor tissues using Expression data; OS, overall survival; UCEC, Uterine Corpus Endometrial Carcinoma; STAD, Stomach Adenocarcinoma; KIRC, Kidney Renal Clear cell Carcinoma; DLBC, Lymphoid Neoplasm Diffuse Large B-cell Lymphoma; BLCA, bladder urothelial carcinoma.
