# Supplementary figures and images for: A novel amino acid metabolism-related gene risk signature for predicting prognosis in clear cell renal cell carcinoma
Source: Front Oncol. 2022 Oct 14;12:1019949. doi: 10.3389/fonc.2022.1019949 (PMC9614380; doi:10.3389/fonc.2022.1019949)

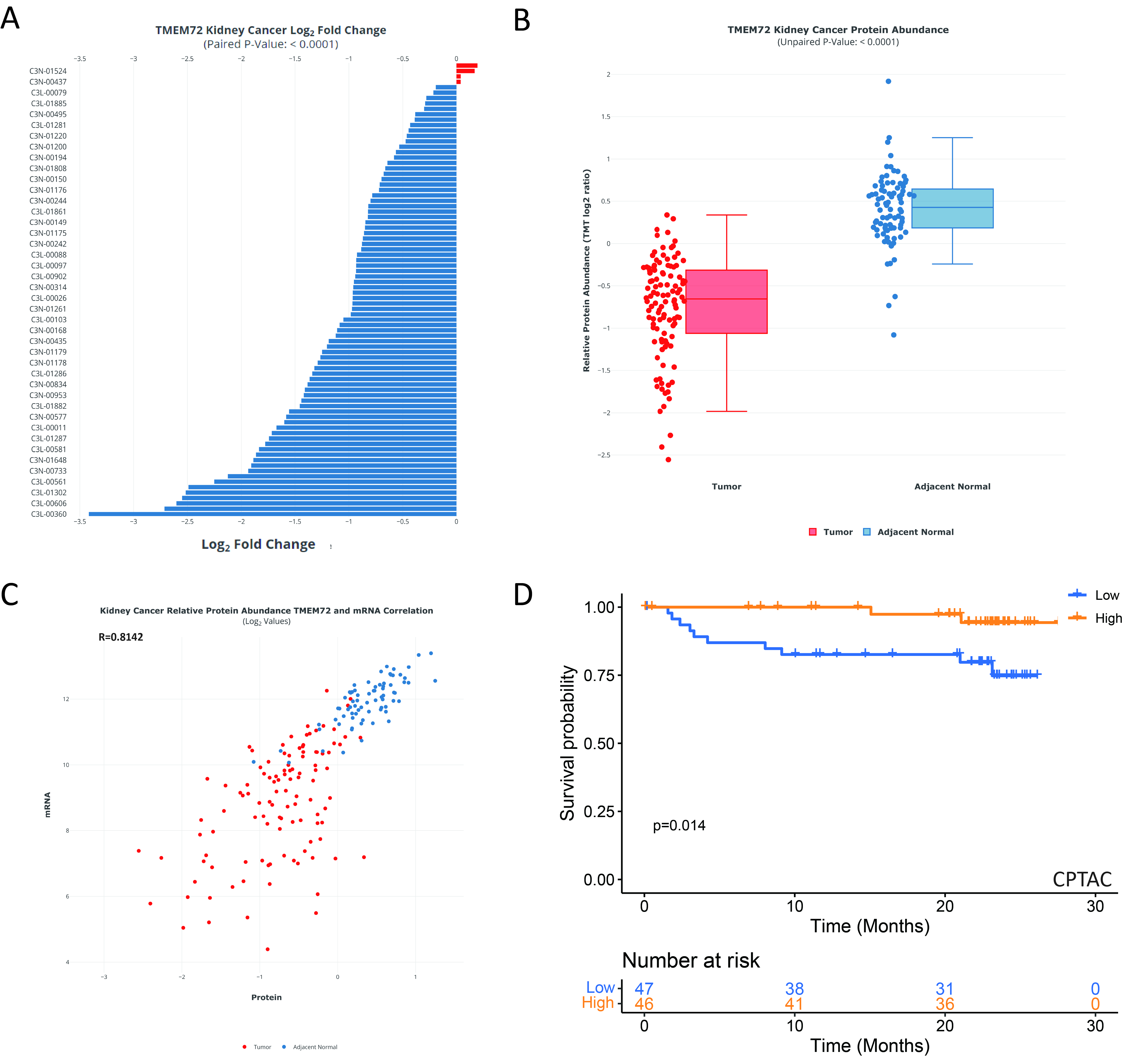

Supplement: Supplementary Figure 1 — TMEM72 protein expression in CPTAC database. (A) The TMEM72 protein expression of paired samples. (B) The TMEM72 protein expression of unpaired samples. (C) The positive correlation between TMEM72 mRNA and protein expression. (R=0.8142) (D) The survival curve showed that downregulation of TMEM72 was a symbol of poor prognosis. (p=0.014).(CPTAC, Clinical Proteomic Tumor Analysis Consortium) [file Image_1.tif]
